# Supplementary material for: Genomic analyses indicate resilience of a commercially and culturally important marine gastropod snail to climate change
Source: PeerJ. 2023 Nov 23;11:e16498. doi: 10.7717/peerj.16498 (PMC10676721; doi:10.7717/peerj.16498)
Supplement: Supplemental Information 1 [file peerj-11-16498-s001.pdf]

# SNP Filtering - PopGen

Population genomic analyses indicate the likely resilience of a commercially and culturally important marine gastropod snail to the effects of climate change

September, 2023

Import packages and data

packages

```
# Clear the global environment
rm(list=ls())

#Load libraries
library(dartR)
library(poppr)
library(ggplot2)
```

Data

```
#Load genlight file into the environment
gl<-gl.load("turbo.gl.all.data.Rdata")

# Let's examine the objective
nLoc(gl)
nInd(gl)
nPop(gl)

#Check the order of individuals' names. If individuals are not ordered by
name, go to the next step
indNames(gl)

#Sort all individuals in pop's alphabetical order
gl <- gl[order(pop(gl)) , ]

indNames(gl)
popNames(gl)

#Details
gl
```

Check genotypes are unique (Ref.: Peter Unmack)

```
#NJ Tree - No filtered
NJ1 <- dist(tab(gl))
tre1 <- nj(NJ1)
```

```
write.nexus(tre1, file = "Phylogram_gl_TM_r2.nex")
plot(tre1)
```

Remove clones

```
gla <- gl.drop.ind(gl, ind.list=c("NAM07","SSI16"))

#Details
gla
```

Summary plots for minor allele frequencies (maf) This script provides summary histograms of MAF for each population in the dataset and an overall histogram to assist the decision of choosing thresholds for the filter function.

```
pdf("gla_report_maf_TM_r2.pdf", height=20, width=20)
gl.report.maf(gla)
dev.off()
```

Filter loci by maf

```
pdf("gla_filter_maf_TM_r1.pdf")
glb <- gl.filter.maf(gla, threshold = 0.03)
dev.off()

#Details
glb
```

Report Call Rates/ Missing Data - loci

SNP datasets generated by DArT have missing values primarily arising from failure to call a SNP because of a mutation at one or both of the the restriction enzyme recognition sites. This script reports the number of missing values for each of several percentiles. The script gl.filter.callrate() will filter out the loci with call rates below a specified threshold.

```
pdf("glb_report_callrate_loc_TM_r1.pdf")
gl.report.callrate(glb, method='loc')
dev.off()
```

Report Call Rates/ Missing Data - individuals

The gl.report.callrate function outputs a table which conveniently shows the number of samples that are retained/excluded for a given threshold. This helps in deciding on a threshold for filtering the dataset.

```
pdf("glb_report_callrate_ind_TM_r1.pdf")
gl.report.callrate(glb, method='ind')
dev.off()
```

Calculate call rate for each locus

```
pdf("glb_filter_callrate_loc_TM_r1.pdf")
glc <- gl.filter.callrate(glb, method="loc", threshold=0.8)
```

```
dev.off()

#Details
glc
```

Calculate call rate for each individual

```
pdf("glb_filter_callrate_ind_TM_r1.pdf")
gld <- gl.filter.callrate(glc, method="ind", threshold=0.8)
dev.off()

#Details
gld
```

Hamming distance - Linkage disequilibrium

```
pdf("gle_Hamming_distance_TM_r1.pdf")
gle <- gl.filter.hamming(gld, threshold=0.2)
dev.off()

#Details
gle
save(gle, file="gle_TM_r1.rdata")
```

Filter Hardy-Weinberg-Equilibrium Filters departure of Hardy-Weinberg-Equilibrium for every loci per population or overall

```
glg <- gl.filter.hwe(gle)

#Details
glg
```

Compare smear plot between original dataset and filtered dataset

```
pdf("gl_original_dataset_TM_r1.pdf")
plot(gla)
dev.off()

pdf("gl_filtered_dataset_TM_r1.pdf")
plot(glg)
dev.off()
```
