# Supplementary material for: Genomic analyses indicate resilience of a commercially and culturally important marine gastropod snail to climate change
Source: PeerJ. 2023 Nov 23;11:e16498. doi: 10.7717/peerj.16498 (PMC10676721; doi:10.7717/peerj.16498)
Supplement: Supplemental Information 2 [file peerj-11-16498-s002.pdf]

SNP Filtering - GEA

Population genomic analyses indicate the likely resilience of a commercially and culturally important marine gastropod snail to the effects of climate change

September, 2023

Import packages and data

packages

```
# Clear the global environment.
rm(list=ls())

#load libraries
library(dartR)
library(poppr)
library(ggplot2)
```

Filter loci by maf

```
#Load the gla file generated with the "SNP Filtering - PopGen" script.
load("gla_TM_r2.rdata")

pdf("gla_filter_maf_TM_r2.pdf")
glb <- gl.filter.maf(gla, threshold = 0.01)
dev.off()

#Details
glb
```

Report Call Rates/ Missing Data - loci}

```
pdf("glb_report_callrate_loc_TM_r2.pdf")
gl.report.callrate(glb, method='loc')
dev.off()
```

Report Call Rates/ Missing Data - individuals

```
pdf("glb_report_callrate_ind_TM_r2.pdf")
gl.report.callrate(glb, method='ind')
dev.off()
```

Calculate call rate for each locus

```
pdf("glb_filter_callrate_loc_TM_r2.pdf")
glc <- gl.filter.callrate(glb, method="loc", threshold=0.8)
dev.off()
```

```
#Details
glc
```

Calculate call rate for each individual

```
pdf("glb_filter_callrate_ind_TM_r2.pdf")
gld <- gl.filter.callrate(glc, method="ind", threshold=0.8)
dev.off()

#Details
gld
```

Hamming distance - Linkage disequilibrium

```
pdf("glf_Hamming_distance_TM_r2.pdf")
gle <- gl.filter.hamming(gld, threshold=0.2)
dev.off()

#Details
gle
```

Identified private alleles

```
#First, transform Genlight to Genind
gi <- gl2gi(gle)
private_alleles(gi)
```

Plot private alleles Reference:
https://grunwaldlab.github.io/poppr/reference/private_alleles.html

```
priv <- private_alleles(gi, report = "data.frame")
ggplot(priv) + geom_tile(aes(x = population, y = allele, fill = count))
```

Drop private alleles

```
glf <- gl.drop.loc(gle, loc.list=c("100012924-65-T/A", "100027429-48-C/T",
"100045382-54-G/C"))

#Details
glf
```

Compare smear plot between original dataset and filtered dataset

```
pdf("gl_original_dataset_TM_r2.pdf")
plot(gl)
dev.off()

pdf("gl_filtered_dataset_TM_r2.pdf")
plot(glf)
dev.off()
```

Compare pre and post filtering

```r
# NJ Tree - No filtered
NJ1 <- dist(tab(gl))
tre1 <- nj(NJ1)
write.nexus(tre1, file = "Phylogram_gl_TM_r2.nex")

# NJ Tree - Filtered
NJ2 <- dist(tab(glf))
tre2 <- nj(NJ2)
write.nexus(tre2, file = "Phylogram_glf_TM_r2.nex")
```
