# Supplementary material for: Genomic analyses indicate resilience of a commercially and culturally important marine gastropod snail to climate change
Source: PeerJ. 2023 Nov 23;11:e16498. doi: 10.7717/peerj.16498 (PMC10676721; doi:10.7717/peerj.16498)
Supplement: Supplemental Information 3 [file peerj-11-16498-s003.pdf]

BayPass

Population genomic analyses indicate the likely resilience of a commercially and culturally important marine gastropod snail to the effects of climate change

September, 2023

References: https://doi.org/10.1038/s41467-021-23027-w, https://doi.org/10.1111/1755-0998.13351

Import packages and data

packages

```
#Clear the global environment
rm(list=ls())

#Load libraries
library(dplyr)
library(lfmm)
library(RSpectra)
library(gdata)
library(vegan)
library(tidyverse)
library(Hmisc)
library(robustHD)
```

Convert genlight to BayPass file

```
#Load genlight object
load("glf_TM.rdata")

# Create INPUT file from filtered DArT data
# Convert genlight object to data frame and then write table

TM_df <- as.data.frame(glf)

write.table(TM_df, "Inputs/Geno/gl2BayPass/TM_012.txt", quote = FALSE)

# Import LONGLAT file, this should have all individuals in the same order as
INPUT file
ind <- glf@ind.names
write.csv(ind, file = "Inputs/Geno/gl2BayPass/TM_ind.csv")

pop <- glf@pop
write.csv(pop, file = "Inputs/Geno/gl2BayPass/TM_pop.csv")

loc.names <- glf@loc.names
```

```
write.csv(loc.names, file = "Inputs/Geno/gl2BayPass/TM_loc.names.csv")

longlat <- read.csv("Inputs/Geno/gl2BayPass/TM_lonlat.csv", header = TRUE)
```

Create function for converting to BayPass file

```
baypass_format <- function(INPUTFILE,LONGLAT,BAYPASSFILE){
  gen<-read.table(INPUTFILE, header = T, row.names=1)
  lonlat = LONGLAT
  allele1 = apply(gen, 2, function(snp) {
    split(snp, lonlat$Population) %>%
      sapply(function(genos) {
        n = sum(!is.na(genos)) * 2
        s = sum(genos, na.rm=T)
        return (s)
      })
  })
  allelecount = apply(gen, 2, function(snp) {
    split(snp, lonlat$Population) %>%
      sapply(function(genos) {
        n = sum(!is.na(genos)) * 2
        s = sum(genos, na.rm=T)
        return (n)
      })
  })
  allelefreq = apply(gen, 2, function(snp) {
    split(snp, lonlat$Population) %>%
      sapply(function(genos) {
        n = sum(!is.na(genos)) * 2
        s = sum(genos, na.rm=T)
        return (s / n)
      })
  })
  allele2<-allelecount-allele1
  whole<-interleave(allele1,allele2)
  baypass<-t(whole)
  write.table(baypass,BAYPASSFILE, col.names = F, row.names = F, sep="\t")
}

# Run baypass conversion function
baypass_format(INPUTFILE = "Inputs/Geno/gl2BayPass/TM_012.txt", LONGLAT =
longlat, BAYPASSFILE = "Inputs/Geno/gl2BayPass/TM_Baypass")
```

In Terminal: Run BayPass (Core model)

Estimate covariance matrix (Ω) of population allele frequencies.

```
#Get Omega matrix
#Run BayPass under the core model mode to generate covariance matrix, repeat
5 times
cd Turbo/BayPass/Core
```

```
module load baypass
g_baypass

for i in 1 2 3 4 5
do
seed=$((1000 + RANDOM % 9999))
echo "$seed"
nohup g_baypass -npop 8 -gfile TM_Baypass -seed $seed -outprefix TM_core.$i -
burnin 2500 -pilotlength 1000 -nthreads 48 &
done
```

In R: Calculate means from initial baypass runs on core model and produce covariance matrix for initital core run with all SNPs

```
myList<- vector(mode = "list", length = 5)

for (i in 1:5) {
  myList[[i]]<-
as.matrix(read.table(paste("TM_core.",i,"_mat_omega.out",sep="")))
}

#mean omega matrix
median <- sapply(1:ncol(myList[[1]]), function(j)
{apply(do.call(cbind,lapply(myList,`[`,,j)), 1, median)})

write.table(median, file=paste0("TM_core.median_mat_omega.out"),
row.names=FALSE, col.names=FALSE, sep="\t")
```

In Terminal: Run BayPass (AUX model)

```
#Run aux model of baypass for assocaitions using created covairance matrix

cd Turbo/BayPass/EA

for i in 1 2 3 4 5
do
seed=$((1000 + RANDOM % 9999))
echo "$seed"
nohup /home/ojhollan/BAM/workingPops/sites/poolFstat/g_baypass -npop 8 -gfile
TM_Baypass -seed $seed -efile TM_env_BP_2.txt -auxmodel -omegafile
TM_core.median_mat_omega.out -outprefix TM_aux.$i -scalecov -burnin 2500 -
pilotlength 1000 -nthreads 48 &
done
```

In R: Calculate means for aux runs and output a csv file with only those above BF20

```
myList<- vector(mode = "list", length = 5)

for (i in 1:5) {
  myList[[i]]<-
```

```
as.matrix(read.table(paste("TM_aux.",i,"_summary_betai.out",sep=""),
header=T))
}

bfmedian <- sapply(1:ncol(myList[[1]]), function(j)
{apply(do.call(cbind,lapply(myList,`[`,,j)), 1, median)})
colnames(bfmedian) <-colnames(myList[[1]])

bfmedian<-as.data.frame(bfmedian)
write.csv(bfmedian, file=paste0("TM_aux_median_summary_betai.out.csv"))

uniq_snpss=bfmedian[,"MRK"][which(bfmedian[,"BF.dB."]>20)]
```
