# Supplementary material for: Genomic analyses indicate resilience of a commercially and culturally important marine gastropod snail to climate change
Source: PeerJ. 2023 Nov 23;11:e16498. doi: 10.7717/peerj.16498 (PMC10676721; doi:10.7717/peerj.16498)
Supplement: Supplemental Information 5 [file peerj-11-16498-s005.pdf]

LFMM2

Population genomic analyses indicate the likely resilience of a commercially and culturally important marine gastropod snail to the effects of climate change

September, 2023

Reference: https://doi.org/10.1111/1755-0998.13351

This code shows how to detect putative genomic signatures of selection using latent factor mixed models (LFMM2).

Import packages and data

packages

```
#Clear the global environment
rm(list=ls())

#Load libraries
library(dartR)
library(LEA)
library(lfmm)
library(dplyr)
library(tibble)
library(stringr)
library(ggplot2)
```

Convert genlight to geno object

```
#Load genlight
load("glf_TM.rdata")

TM.geno<-gl2geno(glf, outpath = getwd())
```

Inferred population structure by estimating individual ancestry coefficients based on sparse non-negative matrix factorisation (SNMF) method implemented in the snmf function in the R package LEA.

```
# SNMF - Inference of individual admixture coefficients
project = NULL
project = snmf("gl_geno.lfmm", K=1:8, ploidy = 2, entropy = T, alpha=100,
rep=100, CPU=4, project = "new")

#To load the project, use:
project = load.snmfProject("gl_geno.snmfProject")
```

Identify ancestral populations (K) by generating an entropy criterion that evaluates the fit of the statistical model to the data using a cross-validation technique.

```
#Plot cross-entropy criterion for each K
K <- summary(project)$crossEntropy %>%
  t() %>%
  as.data.frame() %>%
  rownames_to_column("temp") %>%
  mutate(K = as.numeric(str_extract(temp, "(\\d)+"))) %>%
  select(-temp)

#Choose K for which the function plateaus or increases sharply
ggplot(K, aes(x = K, y = mean)) +
  geom_line(color = "black", size = 0.25 ) +
  geom_segment(aes(x = K, y = min, xend = K, yend = max)) +
  geom_point(shape = 21, size = 3, color = "black", fill = "#CFF09F") +
  geom_point(data=K[1, ], aes(x = K, y = mean), shape = 21, size = 3 , color
= "black", fill = "#0B486D") +
  scale_x_continuous(breaks = seq(1, 28, by = 1)) +
  labs(x = "Number of ancestral populations", y = "Cross-entropy") +
  theme_bw() + theme(panel.border = element_blank(), panel.grid.major =
element_blank(), panel.grid.minor = element_blank(), axis.line =
element_line(colour = "black"))+ theme(legend.position = "none")+
  theme(legend.title=element_text(size=9, face="bold"),
legend.title.align=0.5, axis.line = element_line(colour = 'black', size =
1.25), axis.ticks = element_line(colour = 'black', size = 1.25))
```

Select the best K with the lowest cross-entropy value

```
best = which.min(cross.entropy(project, K = 1))
```

Impute the missing genotypes using snmf project previously created}

```
impute(project, "gl_geno.lfmm",
       method = 'mode', K = 1, run = best)
```

Proportion of correct imputation results

```
x = read.table("gl_geno.lfmm_imputed.lfmm", header=F)
sum(is.na(x)) # 0
```

Run LFMM2

```
#Import Environmental information about populations, no colnames and rownames
E<-read.csv("/Users/simon/Library/CloudStorage/OneDrive-
DeakinUniversity/Collaborations/Turbo_GEA/Inputs/Geo/TM_env_lfmm.csv",
header=F)

#Create directories
#turpval list of SNPs and show the ones that are potential adaptive
dir.create('./turpval/')
dir.create('./prs/')

for (i in 1:length(colnames(E))) { #4 each env variable
```

```
  #### Run LFMM2 #####
  mod.lfmm <- lfmm_ridge(Y = x,
                         X = E[[i]],
                         K = 1) #chose K based on PCA
  #Performs association testing using the fitted model:
  pv <- lfmm_test(Y = x, #x=genotype
                  X = E[[i]], #y=env
                  lfmm = mod.lfmm,
                  calibrate = "gif")
  #Record the calibrated pvalues
  pvalues <- pv$calibrated.pvalue
  hist(pvalues, col = "#E2E2CF")
  length(which(pvalues <0.05))
  write.table(pvalues, paste0("./turpval/tur_",colnames(E)[i],".turpval"),
quote = FALSE)
  #polygenic risk scores
  x.pred <- predict_lfmm(x,E[[i]], mod.lfmm, fdr.level = 0.05, newdata =
NULL) #similar to R2

  #Perform lm on the predicted values vs env (i.e. can snps predict
environment)
  x.lm <- lm(x.pred$pred ~ scale(E[[i]], scale = FALSE))
  #Calculate r2 for the polygenic risk score
  prs <- summary(x.lm)$adj.r.squared #Different to RDA
  write.table(prs, paste0("./prs/tur_",colnames(E)[i],".prs"), quote = FALSE)
}
```

Combine p-values in a single

```
file.list<- list.files("turpval/", pattern=".turpval")

test_read <- read.table(file=paste0("turpval/",file.list[1]), header=T)

#Get the bios
library(stringr)
fl <- word(file.list, 2, sep = "_")
fl2 <- gsub('.turpval', '', fl)

#Read tables in
dataMerge <- data.frame(name=paste0("V",1:dim(test_read)[1]))

for(f in 1:length(file.list)){
  ReadInMerge <- read.table(file=paste0("turpval/",file.list[f]), header=T)
  colnames(ReadInMerge)[1] <- fl2[f]
  dataMerge <- cbind.data.frame(dataMerge,ReadInMerge)
}

all.pv <- dataMerge

write.csv(all.pv, "tur_all_pv.csv", quote=FALSE)
```

```r
all.pv <- read.csv("tur_all_pv.csv") #Check snps are ordered
all.pv <- all.pv[,-1:-2]
head(all.pv)

colnames(all.pv) <- c("SSST","SST","CHLa","EKE","temp_range")

#include name of the snps
ind_snp <- read.table("/Users/simon/Library/CloudStorage/OneDrive-DeakinUniversity/Collaborations/Turbo_GEA/Inputs/Geno/gl2BayPass/TM_loc.names.csv", header = T)

row.names(all.pv) <- ind_snp[,1]

write.csv(all.pv, "./tur_all_pval.csv", quote=FALSE)

all.pv <- data.frame(all.pv)
```

Time to pick the variables to use

```r
all_names <- colnames(all.pv)
sel_bio <- all_names[1:5]
v0 <- NULL

for (i in sel_bio) {
  v0 <- c(v0, which(all.pv[,i] < 0.001))
  cat(length(v0), "\n")
}

uniq_snps <- unique(v0); length(uniq_snps)

x = read.geno("gl_geno.geno")

x_t <- data.frame(t(x)); dim(x_t)

sig_df_1 <- x_t[uniq_snps,]; dim(sig_df_1)

write.csv(sig_df_1, file=paste0("TM_outliers_lfmm2.csv"))

sig_df_1[1:10,1:10]
```

Check SNPs under putatevely selection by environmental variable

```r
nrow(subset(all.pv, SST < 0.001))
nrow(subset(all.pv, CHLa < 0.001))
nrow(subset(all.pv, EKE < 0.001))
nrow(subset(all.pv, temp_range < 0.001))

out.SST <- (subset(all.pv, SST < 0.001))
```

```
out.CHLa <- (subset(all.pv, CHLa < 0.001))
out.EKE <- (subset(all.pv, EKE < 0.001))
out.temp_range <- (subset(all.pv, temp_range < 0.001))

out.SST.df <- data.frame(rownames(out.SST))
out.CHLa.df <- data.frame(rownames(out.CHLa))
out.EKE.df <- data.frame(rownames(out.EKE))
out.temp_range.df <- data.frame(rownames(out.temp_range))

data_names <- c("out.SST.df","out.CHLa.df","out.EKE.df","out.temp_range.df")

for(i in 1:length(data_names)) {          # Head of for-loop
  write.csv2(get(data_names[i]),          # Write CSV files to folder
             paste0(data_names[i],
                    ".csv"),
             row.names = FALSE)
}
```
