# Supplementary material for: Genomic analyses indicate resilience of a commercially and culturally important marine gastropod snail to climate change
Source: PeerJ. 2023 Nov 23;11:e16498. doi: 10.7717/peerj.16498 (PMC10676721; doi:10.7717/peerj.16498)
Supplement: Supplemental Information 7 [file peerj-11-16498-s007.pdf]

Gradient Forest

Population genomic analyses indicate the likely resilience of a commercially and culturally important marine gastropod snail to the effects of climate change

September, 2023

References: https://doi.org/10.1111/ele.12376, https://www.nature.com/articles/s41467-023-36631-9

This code shows how we used Gradient Forest (GF) model to predict areas of high adaptive genetic variation and important environmental drivers across Turbo's geographic distribution. Candidate SNPs identified using LFMM2 and BayPass, and the environmental variables were used as predictors for the gradient forest analysis."

Import packages and data

packages

```
#Clear the global environment
rm(list=ls())

#Load libraries
library(dplyr)
library(tibble)
library(stringr)
library(tidyr)
library(gradientForest)
library(ggplot2)
library(tmap)
library(basicPlotteR)
library(raster)
library(fields)
library(rgdal)
```

Prepare input to make population level allele frequencies

In an excel spreedsheet combined BayPass (TM_outliers_BF20_gm012) and LFMM2 (TM_outliers_lfmm2.csv) results. Get only unique snps and save it as a .txt file in the working directory.

```
#Read genetic matrix in 012 format
x = read.table("/Users/simon/Library/CloudStorage/OneDrive-DeakinUniversity/Collaborations/Turbo_GEA/Inputs/Geno/gl2BayPass/TM_012.txt", check.names=FALSE)

uniq_snpss = read.table("TM_outliers_BP_LF.txt")
```

```
#Remove duplicate detections, if any
uniq_snpss <- uniq_snpss[!duplicated(uniq_snpss$V1),] #NA

x_t <- data.frame(t(x)); dim(x_t) #6852(snps)   206(ind)

x_t <- data.frame(x_t, row.names = paste0(1:6852)); dim(x_t) #6852(snps)
206(ind)

sig_df_1 <- x_t[uniq_snpss,]; dim(sig_df_1) #35(snps)   206(ind)

write.csv(sig_df_1, file=paste0("TM_outliers_BF_LF_gm012.csv"))
```

Calculate population level allele frequencies

```
#Upload Environamntal variables input with rows and columns names
Env<-read.csv("/Users/simon/Library/CloudStorage/OneDrive-
DeakinUniversity/Collaborations/Turbo_GEA/Inputs/Geo/TM_env_lfmm_2.csv",
header=T)[,-3]
head(Env)

#Get allele frequency for each population
tur.af <-  apply(t(sig_df_1), 2, function(snp) {
  split(snp, Env$pop) %>%
    sapply(function(genos) {
      n = sum(!is.na(genos)) * 2
      s = sum(genos, na.rm=T)
      return (s / n)
    })
})

#Check if there are any NAs and convert the NAs to 0
which(apply(tur.af, 2, function(x) any(is.na(x)))) #0
tur.af <- tur.af[ , colSums(is.na(tur.af)) == 0]
```

Run Gradient Forest

```
#Environmental file aggregate
agg_env <- aggregate(. ~ pop, data = Env[,-1], FUN = mean)
all_names <- colnames(Env)
sel_bio <- all_names[3:6]

envGF.tur <- agg_env[,sel_bio]

maxLevel <- log2(0.368*nrow(envGF.tur)/2) #Account for correlations, see
?gradientForest

colnames(tur.af) <- paste0("V", colnames(tur.af))



tur.GF <- gradientForest(cbind(envGF.tur, #Anvironmental variables
```

```
                                tur.af), #Allele freq, arranged by site from
Env. input
                          predictor.vars=colnames(envGF.tur),
                          response.vars=colnames(tur.af),
                          ntree=500, maxLevel=maxLevel,
                          trace=T, corr.threshold=0.50)

summary(tur.GF)
```

R square (min,max,mean)

```
sqar <- tur.GF$imp.rsq
sqar[sqar == 0] <- NA

max(sqar,na.rm = T) #0.2855691

min(sqar,na.rm = T) #0.002792146

mean(sqar,na.rm = T) #0.08470078
```

Overall Importance of Environmental Variables

```
# The predictor overall importance plot shows the mean accuracy importance
and the mean importance weighted by species R2.
most_important <- names(importance(tur.GF))

#Overallimportance in ggplot2
importance(tur.GF)

#        EKE        CHLa         SST temp.range
#0.06129787 0.04311126 0.03566275 0.02327962

tur.GF2=c(0.06129787, 0.04311126, 0.03566275, 0.02327962)
names(tur.GF2)=c("EKE","CHLa","SST","temp.range")
i = data.frame(tur.GF2)
i$var = factor(rownames(i))
i$type = 'Temperature'
i$type[grep('CHLa',i$var)] = 'Productivity'
i$type[grep('EKE',i$var)] = 'Velocity'
i$var = factor(i$var,levels=i$var[order(i[,1])])

ggplot(i,aes(x=var,y=i[,1], fill = type)) +
  coord_flip() + xlab('Environmental variable') + ylab('Proportion of
variance explained') +
  scale_fill_manual(NULL, values=c("#00A1AF","#CC3240","#EDC950")) +
  #theme(legend.position='none',axis.title = element_text(size=16),axis.text=
element_text(size=14) ) +
  geom_bar(stat='identity',
colour=c("#EDC950","#00A1AF","#CC3240","#CC3240"), fill="white", size=2) +
  theme_bw() +
```

```
  theme(panel.border = element_blank(),
        panel.grid.major = element_blank(),
        panel.grid.minor = element_blank(),
        legend.position = "none",
        legend.text = element_text(size=11),
        strip.text.x = element_text(size = 11),
        axis.line = element_line(colour = 'black', size = 1.25),
        axis.ticks = element_line(colour = 'black', size = 1.25),
        axis.text.x = element_text(colour = 'black',size=11),
        axis.text.y = element_text(colour="black",size=11),
        axis.title.x = element_text(colour="black",size=12),
        axis.title.y = element_text(colour="black",size=12),
        strip.background=element_rect(fill="white", linewidth = 2))
```

Split importance

```
plot(tur.GF, plot.type = "S", imp.vars = most_important,
leg.posn = "topright", cex.legend = 0.4, cex.axis = 0.6,
cex.lab = 0.7, line.ylab = 0.9, par.args = list(mgp = c(1.5,
0.5, 0), mar = c(3.1, 1.5, 0.1, 1)))
```

Species Cumulative importance

```
source("gradForestFunctions.R")

par(mgp = c(1.0, 0.1, 0),
    mar = c(2.25, 1, 0.1, 0.5),
    omi = c(0,0.3, 0.1, 0),
    family = "sans", tck = -0.015)
spCumPlot(tur.GF,
          imp.vars = most_important,
          show.species = TRUE,
          legend = FALSE,
          show.overall = FALSE,
          common.scale = TRUE,
          leg.nspecies = 6,
          leg.posn = "topleft",
          frame.plot = FALSE,
          cex.lab = 0.9, cex.legend = 1, cex.axis = 0.8, line.ylab = 0.8)
```

Predictor Cumulative importance

```
plot(tur.GF, plot.type = "C", imp.vars = most_important,
show.species = F, common.sture = T, cex.axis = 0.6,
cex.lab = 0.7, line.ylab = 0.9, par.args = list(mgp = c(1.5,
0.5, 0), mar = c(2.5, 1, 0.1, 0.5), omi = c(0,
0.3, 0, 0)))

#Now in ggplot
preds <- names(tur.GF$overall.imp)
cand.preds.list <- list()
```

```
for(i in preds){
  CU <- cumimp(tur.GF, i)
  cand.preds.list[[i]] <- data.frame(x = CU$x,
                                     y = CU$y,
                                     pred = i,
                                     snps = "cr.cand")
}
cand.preds.df <- do.call(rbind, cand.preds.list)

ggplot() +
  geom_step(data=cand.preds.df, aes(x=x,y=y, colour= pred) ,  linewidth = 1)
+
  scale_colour_manual(values=c("#00A1AF","#EDC950","#CC3240","#CC3240"),
name="pred") +
  facet_wrap(~pred, scale=c("free"), strip.position = "top") +
  ylab("Cumulative Importance") +
  xlab("")+
  theme_bw() +
  theme(panel.border = element_blank(),
        panel.grid.major = element_blank(),
        panel.grid.minor = element_blank(),
        legend.position = "none",
        legend.text = element_text(size=11),
        strip.text.x = element_text(size = 11),
        axis.line = element_line(colour = 'black', size = 1.25),
        axis.ticks = element_line(colour = 'black', size = 1.25),
        axis.text.x = element_text(colour = 'black',size=11),
        axis.text.y = element_text(colour="black",size=11),
        axis.title.x = element_text(colour="black",size=12),
        axis.title.y = element_text(colour="black",size=12),
        strip.background=element_rect(fill="white", linewidth = 2))
```

Species performance rank

```
plot(tur.GF, plot.type = "P", show.names = F, horizontal = F,
cex.axis = 1, cex.labels = 0.7, line = 2.5)
```

Load raster stack to generate prediction

```
av_SST <- brick("/Users/simon/Library/CloudStorage/OneDrive-
DeakinUniversity/Collaborations/Turbo_GEA/Inputs/Geo/Copernicus/sst.mean.2001
_2020.tif")
av_CHLa <- brick("/Users/simon/Library/CloudStorage/OneDrive-
DeakinUniversity/Collaborations/Turbo_GEA/Inputs/Geo/Copernicus/chl.mean.2001
_2020.tif")
av_EKE <- brick("/Users/simon/Library/CloudStorage/OneDrive-
DeakinUniversity/Collaborations/Turbo_GEA/Inputs/Geo/Copernicus/eke.mean.2001
_2020.tif")
temp_range <- brick("/Users/simon/Library/CloudStorage/OneDrive-
DeakinUniversity/Collaborations/Turbo_GEA/Inputs/Geo/Copernicus/sst.mean.rang
e.2001_2020.tif")
```

```r
#Create and Store required extent values (coordinates) in a variable
Extent = c(148.962756, 154.241587, -36.419418, -26.83827)

#Use the new 'Extent' to Crop each raster file to an area covering south
eastern Australia (coordinates already in 'Extent')
av_SST  = crop(av_SST, Extent)
av_CHLa = crop(av_CHLa,Extent)
av_EKE = crop(av_EKE, Extent)
temp_range = crop(temp_range, Extent)

writeRaster(av_SST, "av_SST.tif", overwrite=TRUE)
writeRaster(av_CHLa, "av_CHLa.tif", overwrite=TRUE)
writeRaster(av_EKE, "av_EKE.tif", overwrite=TRUE)
writeRaster(temp_range, "temp_range.tif", overwrite=TRUE)

Env.stack <- stack(av_SST,av_CHLa,av_EKE,temp_range)

#First check names of raster stack
names(Env.stack) #Raster names are not the same as f12_BP_Lf, lets change it!
#"sst.mean.2001_2020"       "chl.mean.2001_2020"       "eke.mean.2001_2020"
"sst.mean.range.2001_2020"

##Lets change stack names
names(Env.stack) <- c("SST","CHLa","EKE","temp.range")

#Check new names
names(Env.stack)
#"SST"        "CHLa"       "EKE"        "temp.range"

Env.stack.grid <- data.frame(SST = values(Env.stack[["SST"]]),
                       CHLa = values(Env.stack[["CHLa"]]),
                       EKE = values(Env.stack[["EKE"]]),
                       temp.range = values(Env.stack[["temp.range"]]))

plot(Env.stack, col=(tim.colors(32)), frame=F, colNA = "black")
```

Biplot

PCA of the transformed grid for plotting in RGB palette. The multi-dimensional biological space can most effectively be represented by taking the principle components of the transformed grid and presenting the first two dimensions in a biplot.

```r
# Transform variables by predicting with GF model
tur.vars <- names(importance(tur.GF))
TM.grid.pre <- predict(tur.GF, Env.stack.grid[complete.cases(Env.stack.grid),
tur.vars]) # need to get rid of NA rows or an error pops up

# TM.PCA of the transformed grid for plotting in RGB palette
```

```
TM.PCA <- prcomp(na.omit(TM.grid.pre), center=TRUE, scale.=FALSE)
summary(TM.PCA)
#Importance of components:
#                           PC1     PC2      PC3      PC4
#Standard deviation     0.03174 0.01057 0.003678 0.001403
#Proportion of Variance 0.88791 0.09843 0.011920 0.001730
#Cumulative Proportion  0.88791 0.98634 0.998270 1.000000

loadings
loadings <- TM.PCA$rotation
#                   PC1         PC2         PC3          PC4
#EKE         0.91921432  0.39296403  0.02447149  0.005045441
#CHLa       -0.02300402  0.02046083  0.34188296  0.939238103
#SST         0.39200155 -0.91119143 -0.10676301  0.068312601
#temp.range  0.02916566 -0.12202785  0.93333748 -0.336362480

TM.a1 <- TM.PCA$x[, 1]
TM.a2 <- TM.PCA$x[, 2]
TM.a3 <- TM.PCA$x[, 3]
TM.r <- TM.a1 + TM.a2
TM.g <- -TM.a2
TM.b <- TM.a3 + TM.a2 - TM.a1
# Scale colors
TM.r <- (TM.r - min(TM.r))/(max(TM.r) - min(TM.r)) * 255
TM.g <- (TM.g - min(TM.g))/(max(TM.g) - min(TM.g)) * 255
TM.b <- (TM.b - min(TM.b))/(max(TM.b) - min(TM.b)) * 255

# Plot predicted genetic turnover in genetic space (TM.PCA biplot)
TM.nvs <- dim(TM.PCA$rotation)[1]
TM.vec <- tur.vars
TM.lv <- length(TM.vec)
TM.vind <- rownames(TM.PCA$rotation) %in% TM.vec
scal <- 25
TM.xrng <- range(TM.PCA$x[, 1], TM.PCA$rotation[, 1]/scal) * 1.1
TM.yrng <- range(TM.PCA$x[, 2], TM.PCA$rotation[, 2]/scal) * 1.1
TM.trns_site <- predict(tur.GF)
TM.PCA.sites <- predict(TM.PCA, TM.trns_site[, tur.vars])
jit <- 0.001

#PC1 VS PC2
knitr::knit_hooks$set(crop = knitr::hook_pdfcrop)
plot((TM.PCA$x[, 1:2]), xlim = TM.xrng, ylim = TM.yrng, xlab = "PC1 (89%)",
ylab = "PC2 (10%)",
     pch = ".", cex = 3, col = rgb(TM.r, TM.g, TM.b, max = 255),
     asp = 1, cex.lab = 1, cex.axis = 1)
rect(par("usr")[1], par("usr")[3],par("usr")[2], par("usr")[4], col =
"white")
par(new = TRUE)
plot((TM.PCA$x[, 1:2]), xlim = TM.xrng, ylim = TM.yrng, xlab = "PC1 (89%)",
```

```
ylab = "PC2 (10%)",
     pch = ".", cex = 3, col = rgb(TM.r, TM.g, TM.b, max = 255),
     asp = 1, cex.lab = 1, cex.axis = 1)
points(TM.PCA.sites[, 1:2],  pch = c(23,21,24,21,22,23,24,25), lwd = 2, cex =
1,
       bg =
c("black","black","black","white","white","white","white","white"),
       col = "black")
text(TM.PCA$rotation[TM.vec, 1]/scal + jit * sign(TM.PCA$rotation[TM.vec,
1]),
     TM.PCA$rotation[TM.vec, 2]/scal + jit * sign(TM.PCA$rotation[TM.vec,
2]),
     labels = TM.vec,  cex = 1, pos = 4)
arrows(rep(0, TM.lv), rep(0, TM.lv),
       TM.PCA$rotation[TM.vec, 1]/scal,
       TM.PCA$rotation[TM.vec, 2]/scal,
       length = 0.1, lwd = 5, angle = 15,
       col = "black")
arrows(rep(0, TM.lv), rep(0, TM.lv),
       TM.PCA$rotation[TM.vec, 1]/scal,
       TM.PCA$rotation[TM.vec, 2]/scal,
       length = 0.1, lwd = 2, angle = 15,
       col = c("#EDC950","#00A1AF","#CC3240","#CC3240"))
#addTextLabels(TM.PCA.sites[, 1],
#              TM.PCA.sites[, 2],
#              rownames(TM.PCA.sites),
#              col.background=rgb(0,0,0, 0.75),
#              cex.label=1, col.label="white")
```

Plot (map) predicted genetic turnover in geographic space

```
#TM.PCA is remvoing NAs, so the TM.vector is shorter than it should and it's
messing up positions. This code introduces back the NA rows in their original
positions
Env.stack.grid$x <- 1:nrow(Env.stack.grid)
Env.stack.grid$nas <- complete.cases(Env.stack.grid)
TM.pcs <- as.data.frame(TM.PCA$x)
TM.pcs$x <- Env.stack.grid$x[Env.stack.grid$nas]
TM.pcs <- merge(TM.pcs, Env.stack.grid, by = "x", all = TRUE)
TM.pcs$r <- TM.pcs$PC1 + TM.pcs$PC2
TM.pcs$g <- -TM.pcs$PC2
TM.pcs$b <- TM.pcs$PC3 + TM.pcs$PC2 - TM.pcs$PC1
TM.pcs$r <- (TM.pcs$r - min(TM.pcs$r, na.rm = TRUE))/(max(TM.pcs$r, na.rm =
TRUE) - min(TM.pcs$r, na.rm = TRUE)) * 255
TM.pcs$g <- (TM.pcs$g - min(TM.pcs$g, na.rm = TRUE))/(max(TM.pcs$g, na.rm =
TRUE) - min(TM.pcs$g, na.rm = TRUE)) * 255
TM.pcs$b <- (TM.pcs$b - min(TM.pcs$b, na.rm = TRUE))/(max(TM.pcs$b, na.rm =
TRUE) - min(TM.pcs$b, na.rm = TRUE)) * 255
TM.raster.r <- TM.raster.g <- TM.raster.b <- Env.stack[[1]]
values(TM.raster.r) <- TM.pcs$r
```

```
values(TM.raster.g) <- TM.pcs$g
values(TM.raster.b) <- TM.pcs$b
TM.rgTMap <- stack(TM.raster.r, TM.raster.g, TM.raster.b)

#Map spatial pattern of predicted genomic composition}
#Load geo coords .csv
TM.coords <- read.csv("/Users/simon/Library/CloudStorage/OneDrive-
DeakinUniversity/Collaborations/Turbo_GEA/Inputs/Geo/TM_env_Pop.csv")

#Load geo coords .shp
TM.coords=readOGR("/Users/simon/Library/CloudStorage/OneDrive-
DeakinUniversity/Collaborations/Turbo_GEA/Inputs/Geo/TM_env_Pop.shp")

#Load NSW polygon
polygon <- readOGR("/Users/simon/Library/CloudStorage/OneDrive-
DeakinUniversity/Collaborations/Turbo_GEA/Inputs/Geo/ne_10m_admin_0_countries
_lakes/ne_10m_admin_0_countries_lakes.shp")

Extent = c(148.962756, 154.241587, -36.419418, -26.73827)
polygon  = crop(polygon, Extent)

tmap_mode("plot")
  tm_shape(TM.rgTMap, raster.downsample = FALSE)+
  tm_rgb()+
  tm_grid(col = "black", lwd = 2, labels.size = 0.7, lines = FALSE) +
  tm_scale_bar(breaks = c(0, 50, 100), text.size = 7, position=c("right",
"bottom"), text.color="white")+
  tm_compass(type="arrow", position=c("left", "bottom")) +
  tm_xlab("Longitude", size = 1)+
  tm_ylab("Latitude", size = 1)+
  tm_shape(polygon) +
  tm_fill(col = "white", border.col="black") +
  tm_borders(lwd = 2) +
  tm_shape(TM.coords) +
  tm_symbols(shape = "location",
          shapes = c(23,21,24,21,22,23,24,25),
          border.col="black",
          col = "white",
          border.lwd=2,
          size = 0.4)+
  tmap_options(check.and.fix = TRUE) +
  tm_layout(0.61, 0.59, 0.61, 0.46, 0.51)
```
